# Giant voltage amplification from electrostatically induced incipient ferroelectric states

**DOI:** 10.1038/s41563-022-01332-z

**Published:** 2022-08-25

**Authors:** Mónica Graf, Hugo Aramberri, Pavlo Zubko, Jorge Íñiguez

**Affiliations:** 1 Materials Research and Technology Department, Luxembourg Institute of Science and Technology (LIST), Esch/Alzette, Luxembourg; 2 London Centre for Nanotechnology and Department of Physics and Astronomy, University College London, London, UK; 3 Department of Physics and Materials Science, University of Luxembourg, Belvaux, Luxembourg

**Keywords:** Ferroelectrics and multiferroics, Atomistic models

## Abstract

Ferroelectrics subject to suitable electric boundary conditions present a steady negative capacitance response^1,2^. When the ferroelectric is in a heterostructure, this behaviour yields a voltage amplification in the other elements, which experience a potential difference larger than the one applied, holding promise for low-power electronics^3^. So far research has focused on verifying this effect and little is known about how to optimize it. Here, we describe an electrostatic theory of ferroelectric/dielectric superlattices, convenient model systems^4,5^, and show the relationship between the negative permittivity of the ferroelectric layers and the voltage amplification in the dielectric ones. Then, we run simulations of PbTiO_3_/SrTiO_3_ superlattices to reveal the factors most strongly affecting the amplification. In particular, we find that giant effects (up to tenfold increases) can be obtained when PbTiO_3_ is brought close to the so-called ‘incipient ferroelectric’ state.

## Abstract (web summary)

Negative capacitance (NC) is a promising route towards low-power electronics. Here, a theory clarifying the connection between NC and voltage amplification is presented, and it is predicted that incipient ferroelectric states that do not necessarily maximize NC can result in a tenfold voltage amplification.

## Main

All materials present a positive global capacitance or dielectric constant on account of thermodynamic stability. Nevertheless, local negative capacitance (NC) states can be obtained in various ways^2,4–13^. Most interestingly, by placing a ferroelectric in contact with a dielectric or non-ideal electrodes^9,14,15^, we can prevent it from reaching its ground state (homogenous polarization), forcing it into a configuration of relatively high energy. Such a frustrated ferroelectric will typically display a steady NC response upon application of an electric field^2,6,7,9,10^. This has been shown in detail for multidomain structures in ferroelectric/dielectric superlattices^4,5,11,16,17^.

**Fig. 1:**
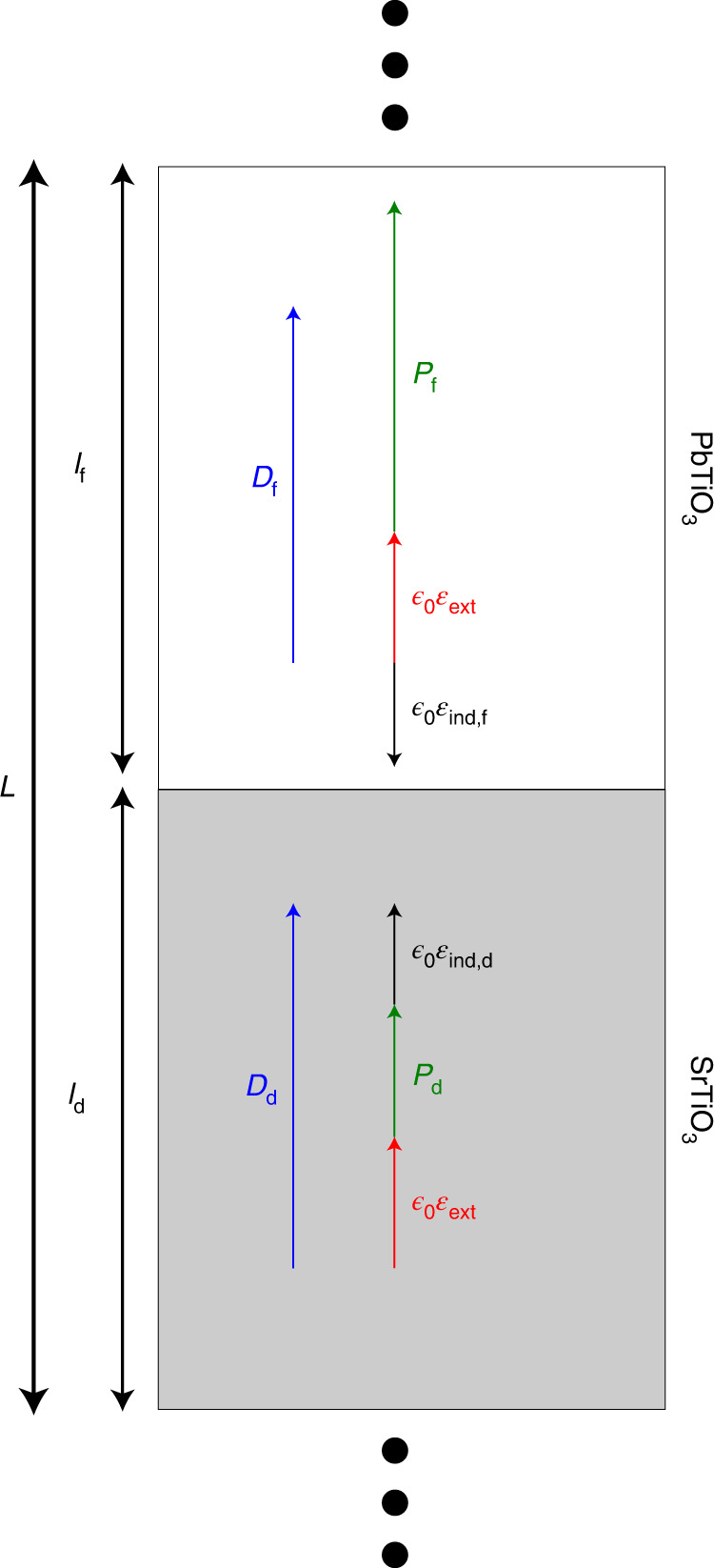
Sketch of a ferroelectric/paraelectric superlattice periodically repeated along the stacking direction. The thicknesses of the ferroelectric and dielectric layers are given by *l*_f_ and *l*_d_, respectively; *L* = *l*_f_ + *l*_d_ is the thickness of the repeated unit. For an arbitrary external field ${{{{\mathcal{E}}}}}_{{{{\rm{ext}}}}}$, and in the absence of free carriers, all layers present the same vertical component of the displacement vector, so that *D*_f_ = *D*_d_. As illustrated in the figure, the displacement *D*_*i*_ of layer *i* involves the layer polarization *P﻿*_*i*_, the field ${{{{\mathcal{E}}}}}_{{{{\rm{ind}}}},i}$ induced in the layer and the external field ${{{{\mathcal{E}}}}}_{{{{\rm{ext}}}}}$.

To understand steady-state NC, consider the superlattice in Fig. 1, where ferroelectric (f) and dielectric (d) layers repeat periodically along the stacking direction *z*. In the absence of free carriers, Maxwell’s first equation dictates ∇ ⋅ *D* = *ρ*_free_ = 0, so the *z*-component of the planar-averaged displacement vector is continuous. We thus have *D* = *D*_f_ = *D*_d_, where *D* is the superlattice displacement while *D*_f_ and *D*_d_ are the layer vectors (*z* subscript omitted for simplicity). Using the definitions in Fig. 1, this yields

1 $$D=P+{\epsilon }_{0}{{{{\mathcal{E}}}}}_{{{{\rm{ext}}}}}={P}_{{{{\rm{f}}}}}+{\epsilon }_{0}{{{{\mathcal{E}}}}}_{{{{\rm{f}}}}}={P}_{{{{\rm{d}}}}}+{\epsilon }_{0}{{{{\mathcal{E}}}}}_{{{{\rm{d}}}}},$$

where *ϵ*_0_ is vacuum permittivity, *P* = *L*^−1^(*l*_f_*P*_f_ + *l*_d_*P*_d_) is the superlattice polarization, ${{{{\mathcal{E}}}}}_{{{{\rm{ext}}}}}$ is the external electric field along *z* and the total field in layer *i* (*i* = f, d) is

2 $${{{{\mathcal{E}}}}}_{i}={{{{\mathcal{E}}}}}_{{{{\rm{ext}}}}}+{{{{\mathcal{E}}}}}_{{{{\rm{ind}}}},i}.$$

Further, as *D* = *D*_*i*_ we have

3 $${{{{\mathcal{E}}}}}_{{{{\rm{ind}}}},i}={\epsilon }_{0}^{-1}\left(P-{P}_{i}\right),$$

which shows that induced fields ${{{{\mathcal{E}}}}}_{{{{\rm{ind}}}},i}$ appear when the local and global polarizations differ. For the f-layer we typically have *P*_f_ > *P*, so that ${{{{\mathcal{E}}}}}_{{{{\rm{ind,f}}}}}$ opposes *P*_f_; this is the so-called ‘depolarizing field’.

Because of the superlattice periodicity, the total voltage associated to the induced fields is null, implying ${l}_{{{{\rm{d}}}}}{{{{\mathcal{E}}}}}_{{{{\rm{i}}}}{\mathrm{nd,d}}}+{l}_{{{{\rm{f}}}}}{{{{\mathcal{E}}}}}_{{{{\rm{ind,f}}}}}=0$. Hence, ${{{{\mathcal{E}}}}}_{{{{\rm{e}}}}{\mathrm{xt}}}$ is the only macroscopic field acting on the system.

To examine the response to a variation of the external field ${\mathrm{d}}{{{{\mathcal{E}}}}}_{{{{\rm{ext}}}}}$, it is useful to introduce a quantity we call the ‘screening factor’, defined for the f-layer as

4 $${\varphi }_{{{{\rm{f}}}}}=\frac{\mathrm{d}{{{{\mathcal{E}}}}}_{{{{\rm{ind,f}}}}}}{\mathrm{d}{{{{\mathcal{E}}}}}_{{{{\rm{ext}}}}}}={\epsilon }_{0}^{-1}\frac{\mathrm{d}\left(P-{P}_{{{{\rm{f}}}}}\right)}{\mathrm{d}{{{{\mathcal{E}}}}}_{{{{\rm{ext}}}}}}=\frac{{l}_{{{{\rm{d}}}}}}{L}\left({\chi }_{{{{\rm{d}}}}}^{\prime}-{\chi }_{{{{\rm{f}}}}}^{\prime}\right).$$

Here we use the primed susceptibilities ${\epsilon }_{0}{\chi }_{i}^{\prime}=\mathrm{d}{P}_{i}/\mathrm{d}{{{{\mathcal{E}}}}}_{{{{\rm{ext}}}}}$, which are all but guaranteed to be positive. (The change in polarization—local or global—will always follow the change in the external field.) The inverse permittivity of the f-layer can then be written as

5 $${\epsilon }_{{{{\rm{f}}}}}^{-1}=\frac{\mathrm{d}{{{{\mathcal{E}}}}}_{{{{\rm{f}}}}}}{\mathrm{d}D}=\frac{\mathrm{d}{{{{\mathcal{E}}}}}_{{{{\rm{ext}}}}}}{\mathrm{d}D}\left(1+{\varphi }_{{{{\rm{f}}}}}\right)={\epsilon }^{-1}\left(1+{\varphi }_{{{{\rm{f}}}}}\right).$$

Further, as detailed in Supplementary Note 1, we can derive the voltage response of the dielectric layer ${{{{\mathcal{A}}}}}_{{{{\rm{d}}}}}$ as

6 $${{{{\mathcal{A}}}}}_{{{{\rm{d}}}}}=\frac{\mathrm{d}{V}_{{{{\rm{d}}}}}}{\mathrm{d}V}=\frac{{l}_{{{{\rm{d}}}}}}{L}\frac{\mathrm{d}{{{{\mathcal{E}}}}}_{{{{\rm{d}}}}}}{\mathrm{d}{{{{\mathcal{E}}}}}_{{{{\rm{ext}}}}}}={L}^{-1}\left({l}_{{{{\rm{d}}}}}-{l}_{{{{\rm{f}}}}}{\varphi }_{{{{\rm{f}}}}}\right).$$

Voltage amplification (VA) corresponds to ${{{{\mathcal{A}}}}}_{{{{\rm{d}}}}} > 1$. This key quantity is fully determined by trivial geometric elements and the screening factor of the f-layer.

We now discuss the dielectric response of a superlattice. Typically the ferroelectric layers will be more responsive than the dielectric ones, so that ${\chi }_{{{{\rm{f}}}}}^{\prime} > {\chi }_{{{{\rm{d}}}}}^{\prime}$. From equation (3), the induced depolarizing field $\mathrm{d}{{{{\mathcal{E}}}}}_{{{{\rm{i}}}}{\mathrm{nd,f}}}$ will oppose $\mathrm{d}{{{{\mathcal{E}}}}}_{{{{\rm{ext}}}}}$, and hence *φ*_f_ < 0. One expects the induced field to be smaller in magnitude than the applied one, so that −1 < *φ*_f_ < 0. It follows that ${\epsilon }_{{{{\rm{f}}}}}^{-1} > 0$ and ${{{{\mathcal{A}}}}}_{{{{\rm{d}}}}} < 1$, a behaviour we may call normal.

Imagine we make the ferroelectric more responsive, for example by varying its temperature to approach the Curie point. We can eventually reach a situation where the induced $\mathrm{d}{{{{\mathcal{E}}}}}_{{{{\rm{ind,f}}}}}$ compensates the applied $\mathrm{d}{{{{\mathcal{E}}}}}_{{{{\rm{ext}}}}}$ (*φ*_f_ = −1), and the voltage drops exclusively in the dielectric layers (${{{{\mathcal{A}}}}}_{{{{\rm{d}}}}}=1$). The ferroelectric effectively behaves as a metal; we call this ‘perfect screening’.

If we keep softening the f-layer so that ${\chi }_{{{{\rm{f}}}}}^{\prime}\gg {\chi }_{{{{\rm{d}}}}}^{\prime}$, we access a regime where the ferroelectric ‘over-screens’^2^: its response is so strong that the induced depolarizing field exceeds the applied one (*φ*_f_ < −1). This yields NC (${\epsilon }_{{{{\rm{f}}}}}^{-1} < 0$) and VA in the dielectric (${{{{\mathcal{A}}}}}_{{{{\rm{d}}}}} > 1$).

Our formulas show that NC and VA can be obtained from the layer polarizations, readily available from the ‘second-principles’ simulations^18–20^ used to explain NC in PbTiO_3_/SrTiO_3_ (PTO/STO) superlattices^4,5^ (Methods). We now use said methods to monitor the dependence of NC and VA on the design variables offered by these materials (layer thickness, epitaxial strain).

We study PTO/STO superlattices where the PTO and STO layers have a thickness of *n* and *m* perovskite cells, respectively, denoted *n*/*m* in the following. We consider *n* and *m* from 3 to 9, and investigate the response to small fields along *z*. We also vary the epitaxial strain *η* between −1% and +3%, choosing the STO substrate as the zero of strain.

We restrict ourselves to low temperatures (formally, 0 K) and work with periodically repeated supercells that are relatively small in plane (8 × 8 perovskite units). This is sufficient to draw conclusions on the behaviour of real materials at ambient conditions.

**Fig. 2:**
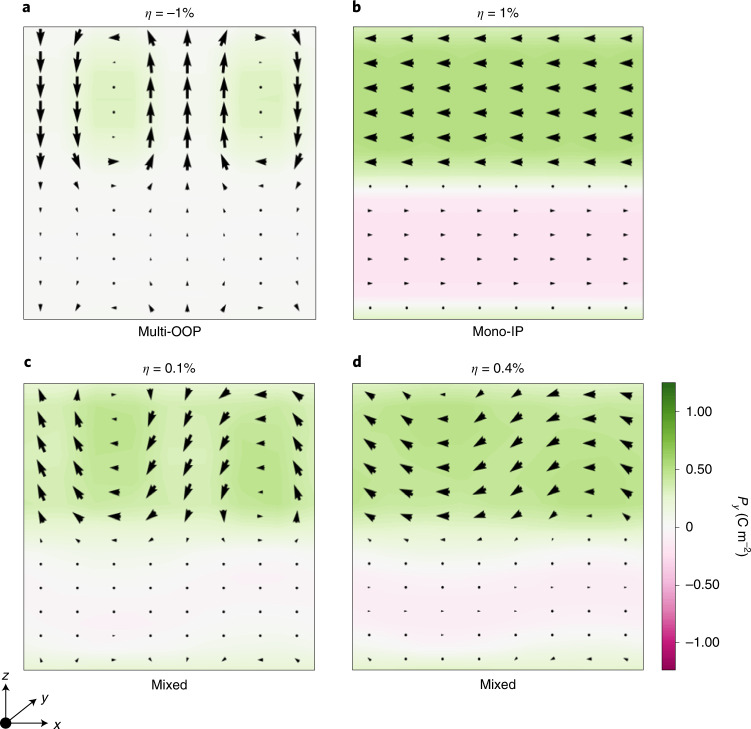
Representative ferroelectric states of PbTiO_3_/SrTiO_3_ superlattices. **a**–**d** Multi-OOP state for *η* = −1% of a 6/6 superlattice (**a**), mono-IP state for *η* = 1% (**b**), and mixed states for *η* = 0.1% (**c**) and 0.4% (**d**). Arrows represent local polarization in the *xz* plane and the colour scale corresponds to the polarization along *y*. The zero of strain corresponds to the lattice constant of bulk STO (3.901 Å). Source data

Let us first recall the main effect epitaxial strain has on PTO/STO superlattices, as obtained from our simulations. Figure 2a shows the ground state of the 6/6 system for *η* = −1%: it presents stripe domains in the PTO layer, with local polarizations along the out-of-plane (OOP) *z* direction. This ‘multi-OOP’ state has been thoroughly studied^4,21–26^.

For large enough tensile strains, we find the PTO layer displays a monodomain state with in-plane (IP) polarization (Fig. 2b). This simulated ‘mono-IP’ configuration is characterized by *P*_*x*_ = *P*_*y*_. In reality^27^, one typically observes the so-called *a*_1_/*a*_2_ multidomain configuration, with local polarizations alternating between *P*_*x*_ and *P*_*y*_. Our monodomain result is a consequence of the relatively small size of the simulation supercell.

Finally, Fig. 2c,d shows states we obtain in some superlattices at intermediate *η* values, where mono-IP and multi-OOP features mix, reminiscent of similar findings in the literature^26,27^. Thus, apart from some non-essential size effects, our simulations capture the evolution of PTO/STO superlattices with epitaxial strain.

**Fig. 3:**
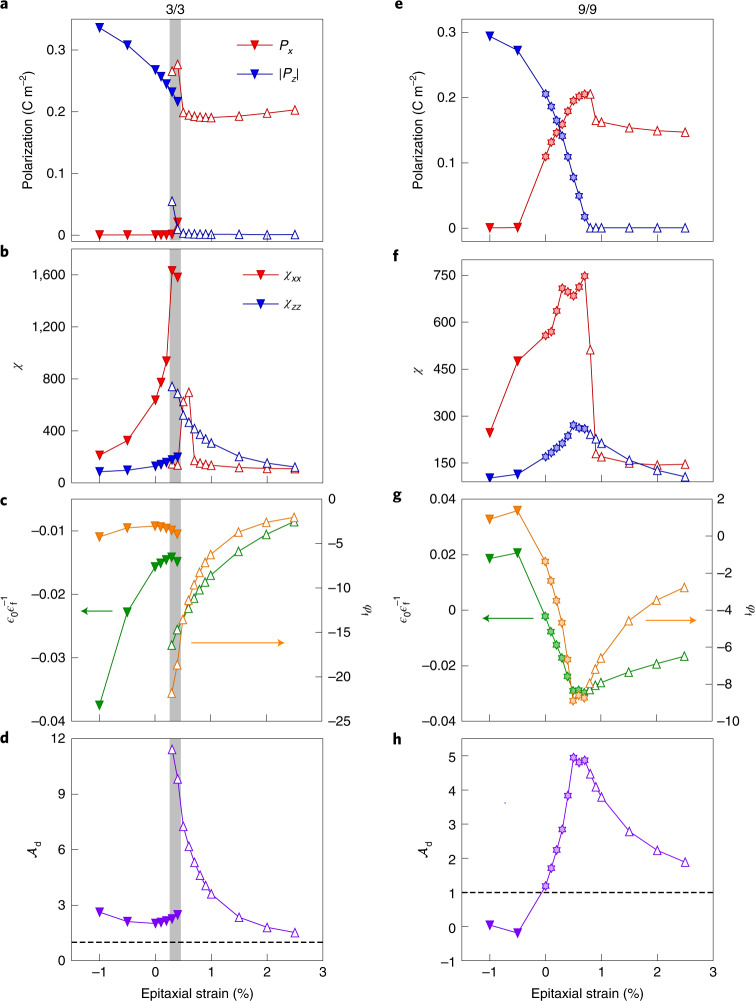
Polar order and response of the PbTiO_3_/SrTiO_3_ superlattices. **a**–**h**, Simulation results for the 3/3 (**a**–**d**) and 9/9 (**e**–**h**) superlattices, as a function of epitaxial strain. Panels **a** and **e** show two superlattice averages of the polarization: $\left|{P}_{z}\right|$ corresponds to averaging the absolute value of the *z*-component of the local polarizations, so as to get a non-zero result in the multi-OOP state; *P*_*x*_ is the direct supercell average of the *x*-component of the local polarizations, where *x* is the modulation direction (perpendicular to the domain walls) in the multi-OOP and mixed states. Panels **b** and **f** show two components, *χ*_*xx*_ and *χ*_*zz*_, of the (global) dielectric susceptibility tensor. Panels **c** and **g** show the inverse permittivity ${\epsilon }_{{{{\rm{f}}}}}^{-1}$ in units of ${\epsilon }_{0}^{-1}$ (left axis) and screening factor *φ*_f_ (right axis) of the ferroelectric layer. Panels **d** and **h** show the voltage ratio ${{{{\mathcal{A}}}}}_{{{{\rm{d}}}}}$ of the dielectric. The grey zone in panels **a**–**d** marks the region where both multi-OOP and mono-IP states are (meta)stable. Dark-coloured down-pointing triangles correspond to multi-OOP states, while we use light-coloured stars for mixed states and empty up-pointing triangles for mono-IP states. Source data

Figure 3a–d shows detailed results for the 3/3 system. At compressive and slightly tensile strains, we get a multi-OOP solution similar to that of Fig. 2a, with ∣*P*_*z*_∣ ≠ 0 and *P*_*x*_ = 0. As *η* increases, we see a transition to the mono-IP phase with ∣*P*_*z*_∣ = 0 and *P*_*x*_ ≠ 0. This transition is discontinuous, both the multi-OOP and mono-IP states being stable at intermediate strains (grey area in the figure).

The global dielectric susceptibility is shown in Fig. 3b. As we increase *η* in the multi-OOP state, we induce a maximum of *χ*_*xx*_, signalling the occurrence of an IP polar instability. In the mono-IP state, it is *χ*_*zz*_ that peaks as *η* decreases, indicating a soft OOP polar mode. The mono-IP state also displays a peak in *χ*_*xx*_ at *η* ≈ 0.6%; this feature, associated to STO and not essential here, is discussed in Supplementary Note 2 and Supplementary Fig. 1.

Figure 3c shows the inverse permittivity (green) and screening factor (orange) of the f-layer. For all considered strains we get ${\epsilon }_{{{{\rm{f}}}}}^{-1} < 0$ and the associated overscreening (*φ*_f_ < −1). Figure 3d shows the corresponding VA in the d layer, which reaches values as high as 12 as the mono-IP state approaches its stability limit. This giant amplification is related to the maximum in *χ*_zz_ (Fig. 3b), in turn connected to the OOP polar instability of the PTO layer. By contrast, the destabilization of the multi-OOP state upon increasing *η*— which involves a *χ*_xx_ anomaly—does not result in any feature in ${\epsilon }_{{{{\rm{f}}}}}^{-1}$ or ${{{{\mathcal{A}}}}}_{{{{\rm{d}}}}}$.

The 9/9 superlattice presents a similar behaviour (Fig. 3e–h), except we find a gradual transformation from multi-OOP to mono-IP, for *η* between 0.0% and 0.8%, with the occurrence of the mixed state mentioned above (Fig. 2c,d). The small jump in *P*_*x*_ around *η* = 0.9% is related to the occurrence of an IP polarization in the STO layer (not relevant here; Supplementary Note 2 and Supplementary Fig. 2).

The 9/9 superlattice displays its largest NC response in this intermediate region, reaching fivefold amplifications at the transition between the mono-IP and mixed states. Interestingly, the multi-OOP state of the 9/9 superlattices shows a peculiar behaviour: see for example ${{{{\mathcal{A}}}}}_{{{{\rm{d}}}}} < 0$ at *η* = −0.5% in Fig. 3h. In this regime, the PTO layer is in a very stable (stiff) multidomain configuration, while the in-plane compression makes STO electrically soft along *z*. Hence, the roles reverse and the STO layer displays NC. (More in Supplementary Note 3.) A similar behaviour has been predicted for BaTiO_3_/SrTiO_3_ superlattices^13^.

**Fig. 4:**
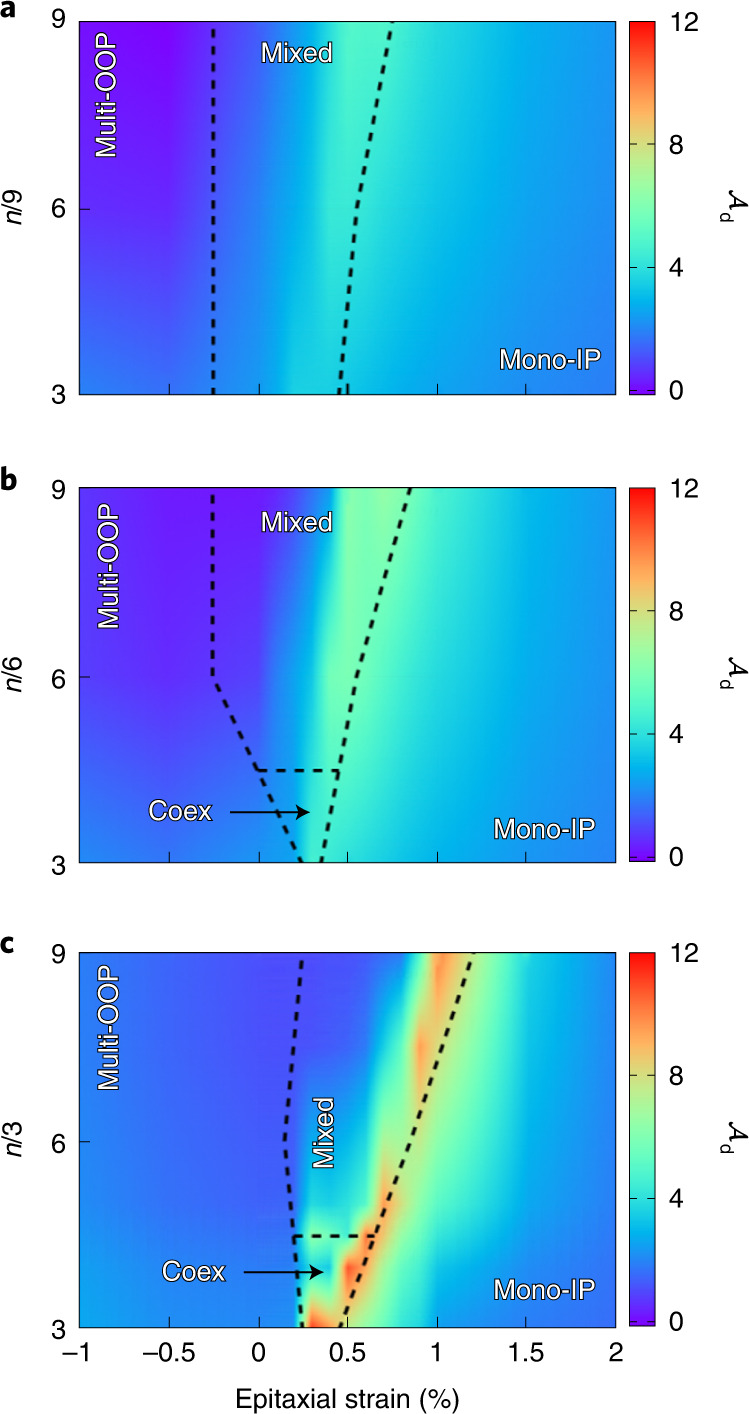
VA maps. **a**–**c** Summary of results for VA in *n*/*m* superlattices: (**a**) *m* = 3, (**b**) *m* = 6 and (**c**) *m* = 9. The colour scale represents the voltage ratio ${{{{\mathcal{A}}}}}_{{{{\rm{d}}}}}$. The lines and labels indicate the stability regions of the states of Fig. 2. In the coexistence region we show the ${{{{\mathcal{A}}}}}_{{{{\rm{d}}}}}$ values corresponding to the mono-IP state. Source data

We run the same study for a large collection of superlattices; Fig. 4a–c summarizes our results. We find the transition region between the multi-OOP and mono-IP states becomes wider for thicker PTO, reflecting the fact that broader layers can accommodate more complex dipole orders, such as the one occurring in the mixed state. (This is consistent with recent observations, for example the occurrence of supercrystals in PbTiO_3_/SrRuO_3_ superlattices with PTO layers above 15 cells^28^.) The mixed state is also favoured by thicker STO layers, a subtle effect probably related to the fact that the stray fields are expelled from the STO layer as it thickens.

Most importantly, Fig. 4 confirms that the strongest amplifications occur at the stability limit of the mono-IP state. It also shows that the multi-OOP region is comparatively unresponsive. Let us now get some insight into the physical underpinnings of these behaviours.

According to equations (4) and (6), VA is determined by the screening factor of the f-layer, which in turn depends on the difference in dielectric response between layers. For example, for the 3/3 superlattice at *η* = 0.3% we get ${{{{\mathcal{A}}}}}_{{{{\rm{d}}}}}\approx 12$, with ${\chi }_{{{{\rm{f}}}}}^{\prime}=765$ and ${\chi }_{{{{\rm{d}}}}}^{\prime}=721$. This ${\chi }_{{{{\rm{f}}}}}^{\prime}$ value may seem small; indeed, the ferroelectric is close to developing an OOP polar instability and one would expect susceptibilities around 10,000 (refs. ^29,30^). By contrast, the computed ${\chi }_{{{{\rm{d}}}}}^{\prime}$ is surprisingly large, as our model for STO yields *χ* = 202 for the pure material. (Our simulated STO is stiff compared with experimental measurements^4^.)

The reason for these surprising ${\chi }_{i}^{\prime}$ susceptibilities can be traced back to electrostatics: all layers respond similarly to an external field, to minimize the depolarizing fields. Thus, we expect ${\chi }_{{{{\rm{f}}}}}^{\prime}\gtrsim {\chi }_{{{{\rm{d}}}}}^{\prime}$. For example, for the 6/6 superlattice at *η* = −1%, which does not display VA, we obtain ${\chi }_{{{{\rm{f}}}}}^{\prime}=96$ and ${\chi }_{{{{\rm{d}}}}}^{\prime}=95$ (Supplementary Fig. 3). Then, when we move to a region of the phase diagram where the f-layer presents an OOP instability, the energy gain associated to the development of d*P*_f_ overwhelms the cost of creating a depolarizing field. Hence, the difference between ${\chi }_{{{{\rm{f}}}}}^{\prime}$ and ${\chi }_{{{{\rm{d}}}}}^{\prime}$ grows a little, sufficient to yield large VA values.

The largest amplifications correspond to the region marking the limit of stability of the mono-IP state. Here the f-layers are in an ‘incipient ferroelectric’ state^2,13^: they are ready to develop an homogeneous OOP polarization whose occurrence is precluded by the presence of the d-layers. Eventually, as we move towards negative *η* values, the multi-OOP polar instability freezes in, leading to either a pure multi-OOP state or a mixed state, and hardening the *z*-polarized ferroelectric soft mode. (This resembles the competition between antipolar and polar orders in antiferroelectrics^31,32^.) This incipient ferroelectric state corresponds to the idealized picture of monodomain NC^2,3^; our results predict a realization of this archetype.

**Fig. 5:**
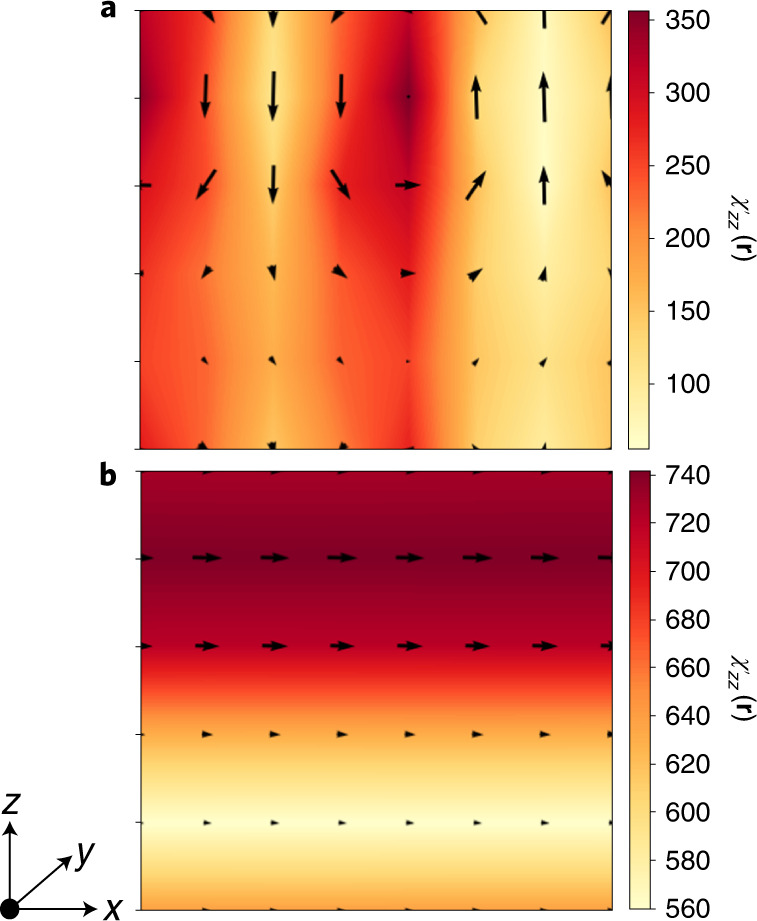
Local responses driving NC and VA. **a**, **b**, Maps of the local dielectric response ${\chi }_{zz}^{\prime}({{{\bf{r}}}})={\epsilon }_{0}^{-1}d P({{{\bf{r}}}})/d {{{{\mathcal{E}}}}}_{{{{\rm{ext}}}}}$, where *P*(**r**) is the position dependent *z*-component of the polarization and the applied field is also along *z*. The results correspond to a particular *xz* plane of the 3/3 superlattice at *η* = 0.4%. (These structures are periodic along *y*.) The arrows represent the local electric dipoles in the *xz* plane at zero field. The shown multi-OOP (**a**) and mono-IP (**b**) states are both stable for this value of *η*. Note that the colour scales differ between panels. Source data

As shown in Fig. 5a and previously reported^4,5^, the NC response of multi-OOP states mainly stems from the strong response (large $\chi ^{\prime}$) of the domain walls. By contrast, the NC of the incipient ferroelectric state comes from the whole f-layer (Fig. 5b), which partly explains its superior VA performance.

Our results thus suggest a strategy to obtain large VA: work with electrostatically induced incipient ferroelectric states that will typically occur at the boundary between IP and OOP phases in ferroelectrics with imperfect screening. Phase boundaries akin to the ones discussed here have been found experimentally in PTO/STO superlattices^27^ and predicted in other ferroelectric/dielectric heterostructures^13^. More specifically, PTO/STO superlattices grown on DyScO_3_ substrates display a coexistence of *a*_1_/*a*_2_ (IP) and vortex (OOP) states at room temperature^27^. Further, the balance between such phases can be tuned by controlling the layer thickness^33^, which should allow stabilization of *a*_1_/*a*_2_ states on the verge of developing an OOP polarization, thus fulfilling the conditions to present strong overscreening in the ferroelectric layer (*φ*_f_ ≪ −1). Those are clear candidates to display giant incipient ferroelectric VA as predicted here. Let us stress that, despite their limitations (low temperature, only monodomain IP states), our simulations capture the physics of the IP-to-OOP transition; thus, we expect our conclusions to apply to experimentally relevant situations.

Additionally, our formulas teach us that ${{{{\mathcal{A}}}}}_{{{{\rm{d}}}}}$ does not depend on the macroscopic permittivity *ϵ*^−1^ (equation (6)), while ${\epsilon }_{{{{\rm{f}}}}}^{-1}$ does (equation (5)). Hence, one can have behaviours such as that of the 3/3 system at *η* = −1% (Fig. 3): a very negative ${\epsilon }_{{{{\rm{f}}}}}^{-1}$ (Fig. 3c) not accompanied by a large ${{{{\mathcal{A}}}}}_{{{{\rm{d}}}}}$ (Fig. 3d). The reason is that this superlattice presents a small *χ*_*zz*_ (Fig. 3b), which yields large *ϵ*^−1^ and $| {\epsilon }_{{{{\rm{f}}}}}^{-1}|$. By the same token, having a globally soft superlattice may result in a modest NC of the f-layer, but this does not necessarily imply a small VA. Hence, for VA purposes, we should not disregard very responsive systems where small values of *ϵ*^−1^ or $| {\epsilon }_{{{{\rm{f}}}}}^{-1}|$ have been observed^4,5,16^. Rather, we must focus on the response difference between ferroelectric and dielectric layers, as captured by the screening factor *φ*_f_.

Finally, let us stress that our conclusions are not restricted to an idealized superlattice. Note that an infinite superlattice is equivalent to a ferroelectric/dielectric bilayer contacted with good electrodes, so there is no net depolarizing field. Further, NC is perfectly compatible with non-ideal electrodes and depolarizing fields^7^; in fact, imperfect screening is at the origin of the effect and can be engineered to induce it^2,6,9^. Hence, we expect our conclusions to apply to real systems whenever the development of an homogeneous polar state is precluded, including field-effect transistors featuring a ferroelectric/semiconductor bilayer.

We hope this work will bring an impetus to the study of NC, shifting the focus to the quantification and optimization of voltage amplification.

## Methods

The second-principles simulations are performed using the SCALE-UP package^18–20^ and the same approach as previous studies of PTO/STO superlattices^4,25,34^. The superlattice models are based on potentials for the pure bulk compounds—fitted to first-principles results^18^—and adjusted for the superlattices as described in ref. ^4^.

We study a collection of *n*/*m* superlattices with layer thicknesses *n*, *m* = {3, 6, 9}. Further, we consider an isotropic epitaxial strain *η* between −1% and 3%, where the STO square substrate (with lattice constant of 3.901 Å) is taken as the zero of strain. Note that STO is a convenient reference on account of the popularity of this substrate in experimental investigations and the fact that it lies on the verge of the OOP-to-IP transformation. Additionally, the STO substrate closely matches the in-plane lattice constant of PTO in the OOP state.

We work with a simulation supercell that contains 8 × 8 perovskite unit cells in the *xy* plane (perpendicular to the stacking direction). In the *z* direction, only one superlattice period is considered. Periodic boundary conditions are assumed.

To find the lowest-energy state of an *n*/*m* superlattice at a given *η* and electric field value, we relax the atomic structure by performing Monte Carlo simulated annealings. During the annealings, all atomic positions and strains are allowed to vary, except for the in-plane strains imposed by the substrate. From the resulting atomic structures, we compute local electric dipoles within a linear approximation (that is, we consider the atomic displacements with respect to the high-symmetry reference structure and multiply them by their corresponding Born charge tensors), as customarily done in second-principles studies^4^.

To compute responses, a small external field of 0.2 MV cm^−1^ is considered. We checked that this field is small enough to obtain susceptibilities and the other relevant quantities within a linear approximation.

We should mention that it is possible to study materials under various electric boundary conditions (that is, at constant electric field^35^ or constant displacement^36^) directly from first principles. Yet, here we adopt a second-principles approach for the sake of computational feasibility. The smallest case simulated in this work (3/3 superlattice) contains 1,920 atoms; the largest (9/9) involves 5,760. Systems of this size remain all but untreatable with today’s first-principles methods.

Finally, let us note that STO is far from being a passive dielectric layer. Indeed, it features structural instabilities of its own: antiphase rotations of the O_6_ groups that are reproduced by our second-principles model^18^ and present in our simulations. Further, the O_6_ tilts compete with an incipient ferroelectric order^37^, and said polar order can be stabilized under epitaxial strain^38^. These effects, and their impact on some of our results, are mentioned in the main text of this article and further addressed in Supplementary Notes 2 and 3. In addition, Supplementary Note 4 and Supplementary Fig. 4 summarize the behaviour of a pure STO film as a function of epitaxial strain, as predicted by our second-principles model.

## Online content

Any methods, additional references, Nature Research reporting summaries, source data, extended data, supplementary information, acknowledgements, peer review information; details of author contributions and competing interests; and statements of data and code availability are available at 10.1038/s41563-022-01332-z.

## Supplementary information

Supplementary Information Supplementary Notes 1–4 and Figs. 1–4.

## Data Availability

Source data for Figs. 2, 3, 4 and 5 are provided with this paper, including the atomic coordinates for structures in Fig. 2. Additional data are available from the authors upon request.

## Source data

Source Data Fig. 2 Structural data, unformatted text.
Source Data Fig. 3 Raw data for figs, table in xlsx file.
Source Data Fig. 4 Raw data for figs, table in xlsx file.
Source Data Fig. 5 Raw data for fig., unformatted text.
