# Supplementary material for: Giant voltage amplification from electrostatically induced incipient ferroelectric states
Source: Nat Mater. 2022 Aug 25;21(11):1252–7. doi: 10.1038/s41563-022-01332-z (PMC9622417; doi:10.1038/s41563-022-01332-z)
Supplement: Supplementary file 1 — Supplementary Notes 1–4 and Figs. 1–4. [file 41563_2022_1332_MOESM1_ESM.pdf]

nature portfolio

https://doi.org/10.1038/s41563-022-01332-z

Supplementary information

Giant voltage amplification from electrostatically induced incipient ferroelectric states

In the format provided by the authors and unedited

Supplementary Materials for "Giant voltage amplification from electrostatically induced incipient ferroelectric states"

Mónica Graf[1], Hugo Aramberri[1], Pavlo Zubko[2] and Jorge Íñiguez[1,3]
[1] *Materials Research and Technology Department, Luxembourg Institute of Science and Technology (LIST), Avenue des Hauts-Fourneaux 5, L-4362 Esch/Alzette, Luxembourg*
[2] *London Centre for Nanotechnology and Department of Physics and Astronomy, University College London, 17–19 Gordon Street, WC1H 0HA London, United Kingdom*
[3] *Department of Physics and Materials Science, University of Luxembourg, Rue du Brill 41, L-4422 Belvaux, Luxembourg*

Contents

Supplementary Note 1: Electrostatic theory of ferroelectric/dielectric superlattices. 1

Supplementary Note 2: In-plane polarization in the $SrTiO_3$ layer under tensile strain. 4

Supplementary Note 3: Relationship between layer response and negative capacitance. 6

Supplementary Note 4: Predicted behavior of a pure $SrTiO_3$ film under epitaxial strain 7

References 7

Supplementary Note 1: Electrostatic theory of ferroelectric/dielectric superlattices.

Let us imagine that we have a periodic stacking of $PbTiO_3$ and $SrTiO_3$ layers, composed of n and m unit cells respectively. Let P_i be the z-component of the polarization of a layer, where $i = \mathrm{f},\mathrm{d}$ is the layer label, as depicted in Fig. 1 of the main text. Let us stress that these polarizations, and all the quantities that appear in our analysis below, must be understood as an average over the corresponding microscopic quantity (e.g., the polarization field) over the volume of the specific layer being considered. Thus, in particular, and most critically, they involve an average over the (infinite) plane perpendicular to the stacking direction of the superlattice. The total electric field of layer i is

$$\mathcal{E}_i = \mathcal{E}_{\rm ext} + \mathcal{E}_{{\rm ind},i} \; , \tag{S.1}$$

where $\mathcal{E}_{\rm ext}$ is the external applied field and $\mathcal{E}_{{\rm ind},i}$ is the layer-specific induced field. $\mathcal{E}_{\rm ext}$ is common to the whole superlattice, and all the local screening effects are captured by $\mathcal{E}_{{\rm ind},i}$. We also define the displacement vector of each layer as $D_i = P_i + \epsilon_0 \mathcal{E}_i$. In absence of free carriers we have $D_i = D$, where D is the displacement vector of the whole superlattice, so we can write:

$$D_i = P_i + \epsilon_0 \mathcal{E}_i = D = P + \epsilon_0 \mathcal{E} \; , \tag{S.2}$$

here, P and $\mathcal{E}$ are the z-component of the superlattice's total polarization and total field, respectively. The latter can be split into external ($\mathcal{E}_{\rm ext}$) and depolarizing ($\mathcal{E}_{\rm dep}$) fields as follows

$$\mathcal{E} = \mathcal{E}_{\rm ext} + \mathcal{E}_{\rm dep} \; . \tag{S.3}$$

The total field is related with the total voltage drop across the superlattice. We have $V = L(\mathcal{E}_{\rm ext} + \mathcal{E}_{\rm dep})$, where $L = l_{\rm f} + l_{\rm d}$ accounts for the thickness of a full period of the superlattice, l_i is the thickness of layer i and V is the voltage difference across L. For periodic boundary conditions, only an (ideal) externally-applied field can yield a net voltate drop across the superlattice, and we have $\mathcal{E}_{\rm dep} = 0$. Therefore, the induced fields must comply with this condition

$$l_{\rm f} \mathcal{E}_{{\rm ind},f} + l_{\rm d} \mathcal{E}_{{\rm ind},d} = L \mathcal{E}_{\rm dep} = 0 \; . \tag{S.4}$$

Working with equations (S.2) and (S.4), we obtain

$$\mathcal{E}_{{\rm ind},i} = \epsilon_0^{-1} \left(P - P_i \right) \tag{S.5}$$

for the induced field in layer i. We are now ready to compute the response to an applied field.

Let us consider the dielectric permittivity of layer i, defined as

$$\epsilon_i^{-1} = \frac{d\mathcal{E}_i}{dD_i} = \frac{d\mathcal{E}_i}{dD} \ , \tag{S.6}$$

where the differentials stand for small variations of the corresponding quantities to a small perturbation of the starting equilibrium state. By substituting equations (S.5), (S.2) and (S.4) into equation (S.6), we can write

$$\epsilon_i^{-1} = \frac{d\mathcal{E}_{\mathrm{ind},i} + d\mathcal{E}_{\mathrm{ext}}}{dP + \epsilon_0 d\mathcal{E}_{\mathrm{ext}}} = \frac{1 + d\mathcal{E}_{\mathrm{ind},i}/d\mathcal{E}_{\mathrm{ext}}}{dP/d\mathcal{E}_{\mathrm{ext}} + \epsilon_0} \ . \tag{S.7}$$

To simplify this expression, let us introduce a quantity that we find very convenient to understand negative capacitance behaviour. We define the "screening factor" of layer i as

$$\varphi_i = \frac{d\mathcal{E}_{\mathrm{ind},i}}{d\mathcal{E}_{\mathrm{ext}}} \ . \tag{S.8}$$

φ_i quantifies how a layer reacts to screen an applied external field. With Eq. (S.5) at hand, Eq. (S.8) can be written as

$$\varphi_i = L^{-1} \left[l_{\mathrm{d}} \ \chi'_{\mathrm{d}} + l_{\mathrm{f}} \ \chi'_{\mathrm{f}} - (l_{\mathrm{d}} + l_{\mathrm{f}}) \ \chi'_i \right] \ , \tag{S.9}$$

where we have used

$$P = L^{-1} \left(l_{\mathrm{f}} P_{\mathrm{f}} + l_{\mathrm{d}} P_{\mathrm{d}} \right) \tag{S.10}$$

and

$$\epsilon_0 \chi'_i = \frac{dP_i}{d\mathcal{E}_{\mathrm{ext}}} \ . \tag{S.11}$$

This is an important relation as it tells us about the ability of the layers to screen the external field and how it is related to the local (primed) susceptibilities. For instance, the screening factor for the f-layer is $\varphi_{\mathrm{f}} = L^{-1} l_{\mathrm{d}} (\chi'_{\mathrm{d}} - \chi'_{\mathrm{f}})$, as expressed in Eq. (4) of the main text.

Going back to the permittivity of a layer i, by replacing Eq. (S.9) in Eq. (S.7), ϵ_i^{-1} can be written as

$$\epsilon_i^{-1} = \frac{1 + \varphi_i}{\epsilon_0 \left(\chi + 1 \right)} = \epsilon^{-1} \left(1 + \varphi_i \right) \ , \tag{S.12}$$

where

$$\epsilon = \frac{dD}{d\mathcal{E}_{\mathrm{ext}}} = \epsilon_0 + \frac{dP}{d\mathcal{E}_{\mathrm{ext}}} = \epsilon_0 (1 + \chi) \tag{S.13}$$

is the permittivity of the whole superlattice, which must always be positive. For the particular case of the ferroelectric layer, Eq. (S.12) reduces to Eq. (5) of the main text.

Let us now turn our attention to the voltage difference in each layer caused by the application of an external field. The voltage drop in layer i can be written as

$$V_i = l_i \mathcal{E}_i \ , \tag{S.14}$$

where we use the convention that a positive voltage is associated to a positive field. Hence, we can differentiate to get

$$dV_i = dl_i \ \mathcal{E}_i + l_i \ d\mathcal{E}_i \approx l_i d\mathcal{E}_i \ , \tag{S.15}$$

where we have neglected the variations of the layer thickness ($dl_i \approx 0$) caused by the change in $\mathcal{E}_{\mathrm{ext}}$. (We have $dl_i = 0$ strictly in all the situations considered in this work, as we restrict ourselves to the linear response of zero-field configurations that are always centrosymmetric – and thus not piezoelectric.) Analogously, for the total applied voltage we have

$$dV \approx L d\mathcal{E}_{\mathrm{ext}} \ . \tag{S.16}$$

The ratio between dV_i and dV, denoted as $\mathcal{A}_i$, tells us how the voltage change in layer i depends on the external voltage. We have

$$\mathcal{A}_i = \frac{dV_i}{dV} = \frac{l_i}{L}\frac{d\mathcal{E}_i}{d\mathcal{E}} = \frac{l_i}{L}\frac{d\left(\mathcal{E}_{\mathrm{ind},i} + \mathcal{E}_{\mathrm{ext}}\right)}{d\mathcal{E}_{\mathrm{ext}}} = \frac{l_i}{L}\left(\varphi_i + 1\right) \ , \tag{S.17}$$

where we have used Eq. (S.8).

Now that we have a compact expression to quantify the voltage ratio in each layer, we are ready to connect the negative-capacitance response of the f-layer and the voltage amplification across the d-layer. If we differentiate Eq. (S.4), still assuming $dl_{\mathrm{i}} \approx 0$, we can write

$$d\mathcal{E}_{\mathrm{ind,d}} = -\frac{l_{\mathrm{f}}}{l_{\mathrm{d}}} d\mathcal{E}_{\mathrm{ind,f}} \ . \tag{S.18}$$

If we replace this into Eq. (S.8) for the dielectric layer, we obtain

$$\varphi_{\mathrm{d}} = \frac{d\mathcal{E}_{\mathrm{ind,d}}}{d\mathcal{E}_{\mathrm{ext}}} = -\frac{l_{\mathrm{f}}}{l_{\mathrm{d}}}\varphi_{\mathrm{f}} \ . \tag{S.19}$$

By replacing Eq. (S.19) into Eq. (S.17), we can express the voltage ratio in the d-layer as a function of the screening factor of the f-layer. We get

$$\mathcal{A}_{\mathrm{d}} = \frac{l_{\mathrm{d}}}{L}\left(1 + \varphi_{\mathrm{d}}\right) = L^{-1}\left(l_{\mathrm{d}} - l_{\mathrm{f}}\varphi_{\mathrm{f}}\right) \ , \tag{S.20}$$

which is Eq. (6) of the main text.

Supplementary Note 2: In-plane polarization in the $SrTiO_3$ layer under tensile strain.

Here we explain how the dielectric properties of the STO layer can produce some features in the dielectric response of the whole system as shown in Fig. 3**b** and 3**e** of the main text.

We show the local dipole distribution of the 3/3 superlattice for small tensile strain in Supp. Fig. 1. In panel **a** we see the local dipoles for the aforementioned superlattice at $\eta = 0.3$ % at zero field; we find that both layers display a polarization along the positive sense of xy direction. If we now apply an external field along x, we get the result in Supp. Fig. 1**b**, both layers undergoing almost no changes. Thus, the associated susceptibility χ_{xx} is small. This is consistent with the small values for this quantity in Fig. 3**b**.

A different behavior can be found in the case with $\eta = 0.5$ %, as displayed in Supp. Figs. 1**c** and 1**d**. In absence of field, the STO layer shows a small polarization opposite to that of the PTO layer. However, an applied field of $\mathcal{E}_x = 0.2$ MV cm^{-1} is enough to reverse the STO polarization, yielding the result in Supp. Fig. 1**d**. This abrupt change in the STO layer results in a peak of χ_{xx} at epitaxial tensile strains around 0.5 % and 0.6 % (see Fig. 3**b** of the main text). Strictly speaking, our applied field is too large to remain in the linear regime in this case; yet, we have kept the result in Fig. 3**b** as it leads to this interesting discussion.

For completeness, we show in panels **e** and **f** of Supp. Fig. 1 the response for higher tensile strains, namely 0.7 %. As in the last case, in absence of field the STO and PTO layers are polarized in opposite senses, but now the polarization of the STO layer is not reversed by our small field of 0.2 MV cm^{-1}. Hence, no substantial change is produced by $\mathcal{E}_x$ and χ_{xx} is small (see Fig. 3**b** of the main text).

Let us also note that the direction of the small STO polarization in the zero-field state, which can be parallel or antiparallel to that of the PTO layer for different values of η, depends on a subtle competition between electrostatic long-range interactions (which favor an antiparallel configuration, resulting from the stray fields created by the xy polarization of the PTO layer) and short-range interactions (which favor the continuity of the xy polarization across the layer interface). As the in-plane polarization of the PTO layer grows (i.e., for larger η values), the electrostatic effect prevails.

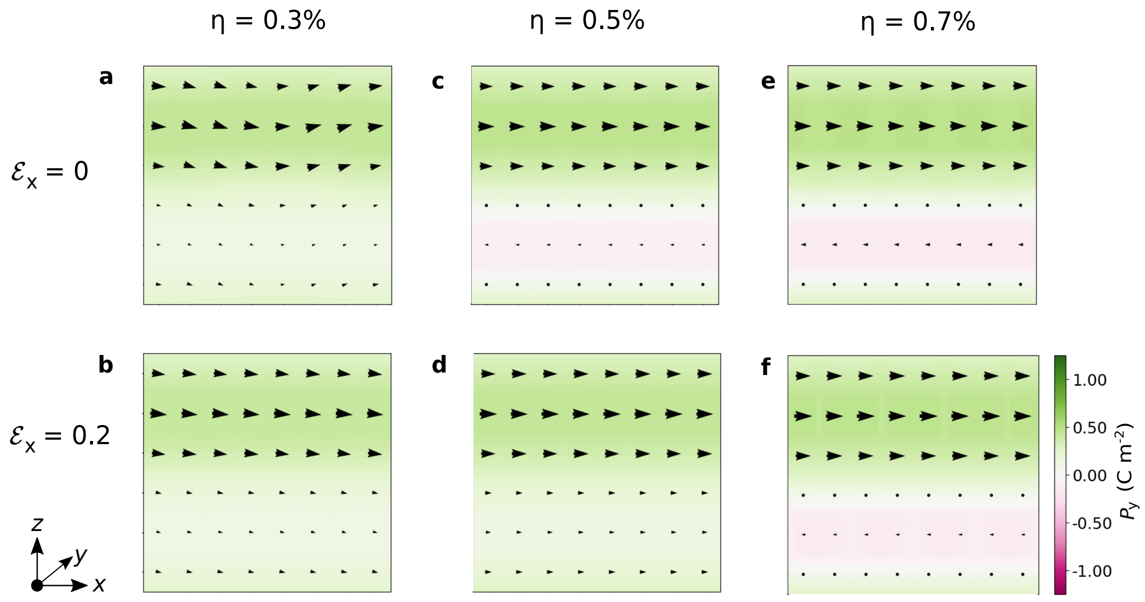

Supp. Fig. 1: **Polarization in the $SrTiO_3$ layer.** Local dipole distributions for the 3/3 superlattice at zero field and at a finite field of 0.2 MV cm^{-1} along x, for different values of tensile epitaxial strain.

Finally, let us comment on the case of the 9/9 superlattice, which displays a decrease of P_x for $\eta \geq 0.9$ % as shown Fig. 3**e** of the main text. We show in Supp. Fig. 2 the x polarization component for the whole system (P_x) and for each layer ($P_{x,\mathrm{d}}$ and $P_{x,\mathrm{f}}$) as functions of strain. It is clear from the figure that the jump in the average polarization is caused by an increase in the magnitude of the polarization in the STO layer. For strains higher than 0.9 %, the d-layer starts to have a polarization comparable in magnitude to that of the f-layer, but opposed to it. As a result, the global average polarization is affected accordingly.

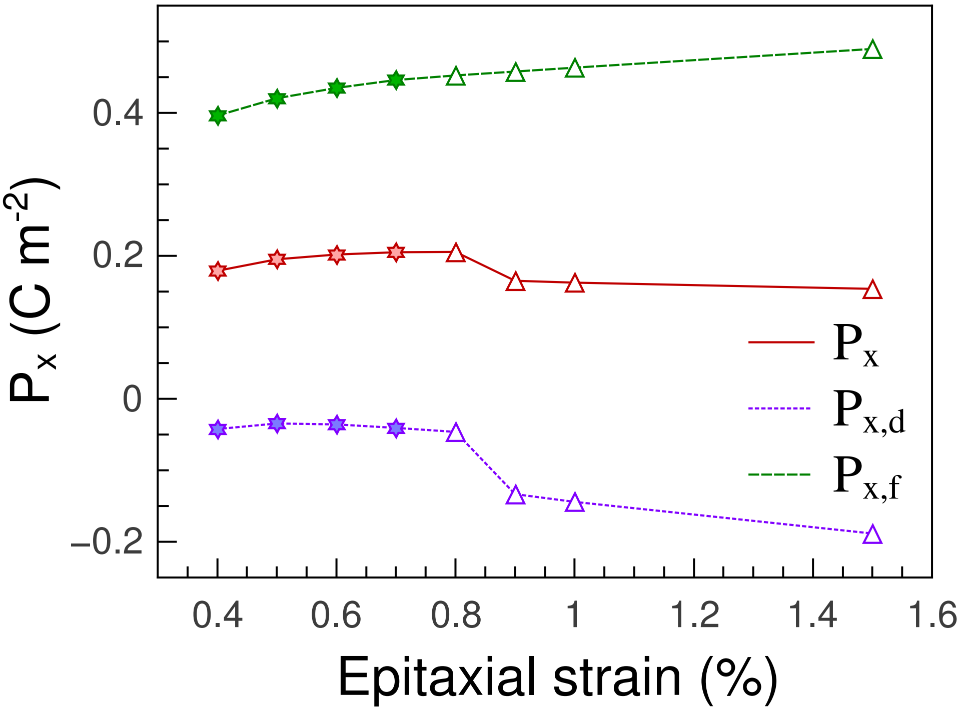

Supp. Fig. 2: **Layer-resolved in-plane polarization.** Polarization along x for the 9/9 superlattice as a function of epitaxial strain. We show the global value (P_x) as well as the result for individual layers. The symbols are as in Fig. 3 of the main text.

Supplementary Note 3: Relationship between layer response and negative capacitance.

From our electrostatic theory of ferroelectric/dielectric superlattices we know that negative-capacitance behavior is related with the different response of each layer to an external field (see Eqs. 4-6 of the main text). Let us now exemplify this point by showing how the layer-resolved primed susceptibilities – i.e. $\chi'_{\rm f}$ and $\chi'_{\rm d}$ – evolve with epitaxial strain. More precisely, Supp. Fig. 3 shows results for the 6/6 superlattice. For $\eta \leq 0$ %, the f-layer displays a positive capacitance ($\varphi_{\rm f} > -1$). When this happens, as can be seen in panel **a**, both layers respond very similarly to the applied field ($\chi'_{\rm f} \approx \chi'_{\rm d}$). By contrast, for higher tensile strains the f-layer enters the negative-capacitance regime with a small-but-sizeable difference between $\chi'_{\rm f}$ and $\chi'_{\rm d}$.

As a final remark, let us note that, while negative capacitance is usually found in the ferroelectric PTO layer, under certain conditions the dielectric STO can be the one presenting the effect. For instance, Fig. 3**h** of the main text shows that a negative voltage ratio ($\mathcal{A}_{\rm d} < 0$) appears at $\eta = -0.5$ % in the 9/9 superlattice. In this case we have $\chi'_{\rm f} = 112$ and $\chi'_{\rm d} = 115$, which yields $\varphi_{\rm d} = -1.4$ and $\epsilon_{\rm d}^{-1} = -0.003\,\epsilon_0^{-1}$ for the d-layer. The explanation for this behavior is a simple one: in this compressive regime, the multidomain ferroelectric order is well developed in PTO, which renders electrically-stiff f-layers that act as dielectrics. By contrast, the in-plane compression drives an out-of-plane polar instability in the STO layers, which then become ferroelectric. A similar behavior was first predicted in Ref. 1 for $BaTiO_3/SrTiO_3$ superlattices.

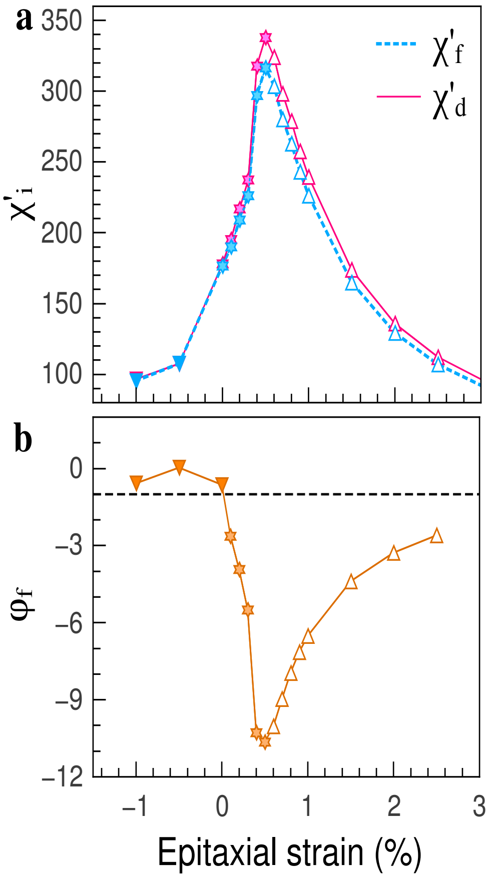

Supp. Fig. 3: **Insight into the screening factor.** Layer-resolved primed susceptibilities (**a**) and screening factor of the f-layer (**b**) for the 6/6 superlattice as functions of epitaxial strain. Recalling Eq. 4 of the main text, the differenciated layer responses explains the occurrence of overscreening and negative capacitance in the f-layer. The symbols are as in Fig. 3 of the main text.

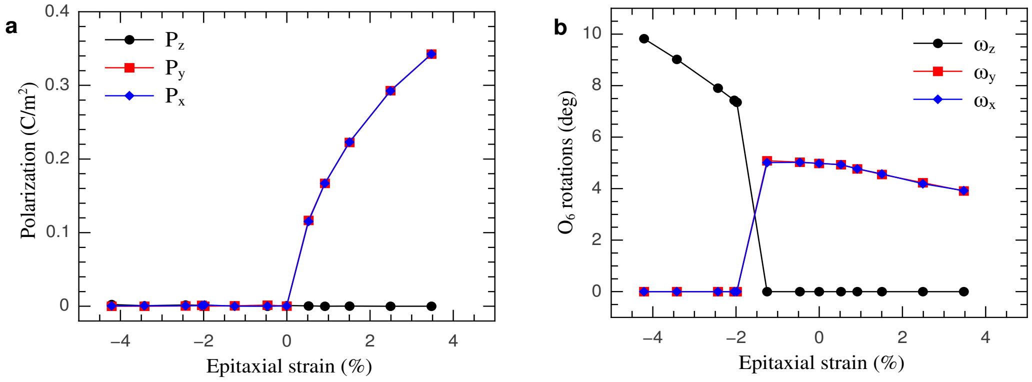

Supp. Fig. 4: **Predicted behavior of pure $SrTiO_3$ films.** Polarization (a) and O_6 antiphase rotations (b) of a pure $SrTiO_3$ film, at 0 K and as a function of epitaxial strain, as predicted by the second-principles model used in this study. The zero of strain corresponds to a square lattice with a lattice constant of 3.901 Å

Supplementary Note 4: Predicted behavior of a pure $SrTiO_3$ film under epitaxial strain

In order to have a better insight into the effect strain has on the STO layer, we also show here the response of a pure STO film under the same mechanical conditions. We use the same second-principles model for $SrTiO_3$; also as before, the zero of epitaxial strain corresponds to a square substrate with lattice constant of 3.901 Å. The results are shown in Supplementary Fig. 4.

Upon moderate tensile strain, our model predicts an in-plane electric polarization to appear. More precisely, this polarization along the xy direction (on panel a) coexists with antiphase O_6 rotations about the xy axis (on panel b). Its occurrence is certainly compatible with the experimental observations[2].

By contrast, we find that no polarization appears upon in-plane compression. In this case, the O_6-rotational order becomes very strong and precludes (according to our model) the onset of the out-of-plane polar instability. Again, this seems compatible with the fact that, experimentally, the observation (or absence) of a polarization upon compression remains an open question[2–4].

Here we should note that our model for $SrTiO_3$ was fitted to reproduce first-principles data corresponding to equilibrium situations at zero external stress. Hence, we do not expect it to be very accurate to describe strongly strained states, and in particular the effect of such strains on the subtle competition between octahedral rotations and ferroelectricity that occurs in this compound. Nevertheless, the behavior it predicts seems physically sound and compatible with experiment.

Let us also note that the behavior of the $SrTiO_3$ layers in the superlattices can be expected to differ from what is shown in Supplementary Fig. 4. Most importantly, since the $PbTiO_3$ layers do not present oxygen-octahedral rotations, their occurrence in the STO layer is partly hampered, which should facilitate the onset of polar instabilities.

Nevertheless, these differences do not seem essential to explain the dielectric response of the STO layers in the superlattice, which is addressed in some detail in the main text as well as in Supplementary Note 2.

[1] R. Walter, S. Prosandeev, C. Paillard, and L. Bellaiche, npj Computational Materials **6**, 186 (2020).

[2] J. H. Haeni, P. Irvin, W. Chang, R. Uecker, P. Reiche, Y. L. Li, S. Choudhury, W. Tian, M. E. Hawley, B. Craigo, et al., Nature **430**, 758 (2004).

[3] D. Nuzhnyy, J. Petzelt, S. Kamba, X. Martí, T. Čechal, C. M. Brooks, and D. G. Schlom, Journal of Physics: Condensed Matter **23**, 045901 (2011).

[4] T. Yamada, B. W.-v. Eerd, O. Sakata, A. K. Tagantsev, H. Morioka, Y. Ehara, S. Yasui, H. Funakubo, T. Nagasaki, and H. J. Trodahl, Phys. Rev. B **91**, 214101 (2015).
